# A retrospective study of Newcastle disease in Kenya

**DOI:** 10.1007/s11250-019-02059-x

**Published:** 2019-09-10

**Authors:** Auleria A. Apopo, Henry M. Kariithi, Leonard O. Ateya, Yatinder S. Binepal, Jane H. Sirya, Thomas D. Dulu, Catharine N. Welch, Sonia M. Hernandez, Claudio L. Afonso

**Affiliations:** 1 Directorate of Veterinary Services, State Department for Livestock, Ministry of Agriculture, Livestock, Fisheries and Irrigation, Private Bag-00625, Nairobi, Kenya; 2 Biotechnology Research Institute, Kenya Agricultural and Livestock Research Organization, P.O Box 57811, Kaptagat Road, Loresho, Nairobi, 00200 Kenya; 3 Exotic and Emerging Avian Viral Diseases Research Unit, Southeast Poultry Research Laboratory, Agricultural Research Service, U.S. Department of Agriculture, US National Poultry Research Center, 934 College Station Road, Athens, GA 30605 USA; 4 Department of Biochemistry, Jomo Kenyatta University of Agriculture and Technology, P.O. Box 62000, Nairobi, 00200 Kenya; 5 Warnell School of Forestry and Natural Resources and The Southeastern Cooperative Wildlife Disease Study at the College of Veterinary Medicine, University of Georgia, Athens, GA 30602 USA

**Keywords:** Avian orthoavulavirus 1, Newcastle disease, Diagnostics, Poultry production system

## Abstract

Newcastle disease (ND) is a major constraint to Kenya’s poultry production, which is comprised of approximately 80% indigenous chickens (ICs; caged and free-range system) and 20% exotic chickens (intensive system). This study analyzed cases reported as suspected ND in Kenya between 2005 and 2015. Of the suspected 332 ND reported cases from the three production systems in 27 locations within six Kenyan Agro-Ecological Zones (AEZs), 140 diagnosed as infected with avian orthoavulavirus 1 (AOaV-1; formerly Newcastle disease virus) were present in every year in all AEZs. The numbers of AOaV-1-positive cases differed significantly (*p* < 0.05) between the production systems across the years depending on the season, climate, and location. In the free-range system, both ambient temperatures and season associated significantly (*p* = 0.001 and 0.02, respectively) with the number of cases, while in the intensive and caged systems, the positive cases correlated significantly with season and relative humidity, respectively (*p* = 0.05). Regardless of the production systems, the numbers of clinically sick birds positively correlated with the ambient temperatures (*r* = 0.6; *p* < 0.05). Failure to detect AOaV-1 in 58% of the ND cases reported, and mortalities exceeding the observed numbers of clinically sick birds suggest deficiencies in the current ND reporting and diagnostic system. Intensive farmers were the slowest in reporting the cases and diagnostic deficiencies were most evident by failure to test the exposure of ICs to natural infection with AOaV-1 and for the AOaV-1-negative cases lack of testing for other pathogens and/or AOaV-1 variants. This study indicates a need for improved surveillance and diagnostics in Kenyan domestic poultry.

**Electronic supplementary material:**

The online version of this article (10.1007/s11250-019-02059-x) contains supplementary material, which is available to authorized users.

## Introduction

Newcastle disease (ND) is an important infectious disease of domestic poultry caused by virulent strains avian orthoavulavirus 1 (AOaV-1; formerly avian paramyxovirus-1; *Paramyxoviridae* family) (Kuhn et al. 2019). AOaV-1 contains a linear non-infectious negative single-stranded RNA (ssRNA) genome encoding at least six proteins (de Leeuw and Peeters 1999). Currently, complete genomes of over 15 AOaV-1 serotypes are available; however, the most-studied is AOaV-1 (formerly Newcastle disease virus; NDV), which can be either virulent or avirulent in chickens (Gogoi et al. 2017). The first confirmed ND outbreak in domestic poultry occurred in the mid-1920s in India, Sri Lanka, Indonesia, Korea, Japan, and, England (Leighton and Heckert 2007; Alexander 2009). Since then, several devastating ND panzootics have recurred (Miller et al. 2015; Dimitrov et al. 2016). Occurrence of ND outbreaks long after AOaV-1 emergence (Lomniczi et al. 1998) implicates the existence of reservoir hosts including wild aquatic wild birds and domestic poultry, Columbiform birds, and double-crested cormorants (Snoeck et al. 2013; Rehmani et al. 2015; Cross et al. 2013; Brown and Bevins 2017). Carrier birds, continuous introduction of susceptible and/or exotic bird species, conducive climates, and AOaV-1 heterogeneity contribute to the maintenance of AOaV-1 in poultry populations (Awan et al. 1994).`

AOaV-1 is relatively stable in nature where it can remain infectious for several weeks at low temperatures, and if protected by bird’s feathers and within eggs, the virus may survive for over 250 days (Leighton and Heckert 2007). In chickens and susceptible species of wild birds, the highly contagious forms of AOaV-1 often result in high mortalities, which can exceed 50% in susceptible flocks (Alexander 2000; Alexander et al. 2012). In rural poultry populations, AOaV-1 pathogenesis depends on factors such as host species, age structure, immune status, viral loads, and viral heterogeneity (Alexander and Senne 2008). Under intensive poultry production systems, especially in the non-immune flocks, the introduction of virulent AOaV-1 can cause severe ND outbreaks.

The poultry industry in Kenya is dominated by indigenous chickens (ICs) (Kemboi et al. 2013), i.e., “non-descript crosses of Asiatic meat and game types, Mediterranean egg-types and Bantams of various origins,” which are mostly kept under free-range or caged management (Kingori et al. 2010; Magothe et al. 2012). Frequent ND outbreaks despite intensive vaccinations suggest endemicity of the disease in Kenya (Lichoti 2000; Njagi et al. 2010; Ashraf and Shah 2014). A recent study suggested possible cross-border spread of velogenic AOaV-1 between Kenya and the neighboring Uganda through live-bird trade (Ogali et al. 2018). AOaV-1 infections may persist asymptomatically within poultry populations until discontinuation or failure of vaccinations, or because of opportunistic infections with other pathogens or parasites. It should be noted that a locally available live-attenuated vaccine (AVIVAX-L™ and AVIVAX-F™) is used for vaccinations against AOaV-1 in Kenya. This thermostable I-2 vaccine (derived from lentogenic LaSota/F strains) is administered in three doses (as eye drops): first dose is when the birds are 4 days to 3 weeks old, second dose at the age of 4–8 weeks, and the last dose at the age of 8–18 weeks. To overcome the problem of continuous ND outbreaks, some commercial farmers often use different combinations of live and/or inactivated vaccines, which are based on older AOav-1 genotypes, and have been reported to be inefficient in reducing viral replication and shedding (Rehmani et al. 2015). Furthermore, even with availability of these vaccines, vaccination rates outside of commercial farming are low in in the country, a trend that has been reported in Tanzania (Campbell et al. 2018). There is insufficient data to account for the causes of ND outbreaks in Kenya, especially in vaccinated commercial flocks. In addition, technology for definitive definition of cases has not been available until recently.

The current study analyzed ND cases reported over an 11-year period (2005–2015) using data derived from 27 locations within six Agro-Ecological Zones (AEZs) in Kenya (Table 1). The primary objective of the study was to provide baseline data for improved diagnosis, surveillance, and control of ND in Kenya. Due to the lack of absolute identification methods in Kenya during the reported period, ND cases are defined as follows. A “ND suspect case” refers to samples obtained from birds showing clinical signs consistent with ND (Alexander 2000). “Likely AOaV-1-positive case” consisted of samples that although lacking definitive virus isolation and sequence identification fulfilled other criteria of virulent AOaV-1 infection. This included (i) post-mortem (PM; necropsy) lesions suggestive of ND, (ii) diagnosis by virus isolation in embryonated chicken eggs, (iii) hemagglutination inhibition (HI) assay, and (iv) real-time reverse transcription polymerase chain reaction (rRT-PCR) assay (Alexander 2012; Wise et al. 2004). AOaV-1-negative cases were samples that although initially were reported to show clinical signs of ND failed the PM or other confirmatory tests. Since little information about ND is currently available in Kenya, the data presented in this paper are a useful resource for future studies on characterization of AOaV-1 strains/isolates and the epidemiology, surveillance, and diagnostics of the disease in the country.

**Table 1:** Summary of the filtered data on the 332 ND cases reported from 2005 to 2015 period that were analyzed in the current study. The cases in this table are categorized based on the season, production system, and the AEZs (see details in the manuscript text)

Category		2005	2006	2007	2008	2009	2010	2011	2012	2013	2014	2015	Total	% of total
Season of the year	Dry (Jan–Feb)	0	13	2	5	3	3	0	8	2	1	1	38	11.45
	Long-rains (Mar–May)	1	81	7	5	4	8	10	10	1	1	3	131	39.46
	Cold (Jun–Oct)	9	34	18	8	2	18	12	9	3	1	3	117	35.24
	Short-rains (Nov–Dec)	5	8	6	0	1	10	3	8	3	0	2	46	13.86
Category of submitter^1^	DVO	1	32	3	1	0	1	2	2	0	0	0	42	12.65
	Owner (farmer)	3	46	14	10	1	17	11	10	8	2	5	127	38.25
	Private Vet	0	8	0	1	2	0	0	1	0	1	0	13	3.92
	RVIL	11	39	15	6	6	21	11	22	1	0	4	136	40.96
	VEEU	0	11	1	0	1	0	1	0	0	0	0	14	4.22
Type of bird	Chicken	14	124	32	18	10	39	25	35	9	3	9	318	95.78
	Duck	0	7	0	0	0	0	0	0	0	0	0	7	2.11
	Turkey	1	2	1	0	0	0	0	0	0	0	0	4	1.20
	Goose	0	3	0	0	0	0	0	0	0	0	0	3	0.90
Poultry production system	Caged	0	12	2	1	2	8	1	6	1	0	4	37	11.14
	Free-range	6	46	13	5	2	12	6	3	1	1	0	95	28.61
	Intensive	9	68	18	12	6	19	16	26	7	2	4	187	56.33
	“Unknown” *	0	10	0	0	0	0	2	0	0	0	1	13	3.92
Agro-ecological zone (AEZ)	Zone II: (upper highlands)	1	32	7	3	0	9	2	3	1	3	2	63	18.98
	Zone III: (lower highlands)	9	28	12	5	7	22	11	20	1	0	3	118	35.54
	Zone IV: (upper midlands)	2	57	11	9	3	8	11	9	7	0	3	120	36.14
	Zone V: (lower midlands)	0	6	1	0	0	0	1	0	0	0	1	9	2.71
	Zone VI: (inner lowlands)	0	0	1	1	0	0	0	1	0	0	0	3	0.90
	Zone VII: (Coastal lowlands)	3	13	1	0	0	0	0	2	0	0	0	19	5.72
Total numbers of reported ND cases		15	136	33	18	10	39	25	35	9	3	9	332	332
AOaV-1 test results	Negative	12	81	25	14	8	14	9	20	2	0	3	188	56.63
	Probable-positive	3	55	8	4	2	25	16	15	7	3	6	144	43.37

^1^*DVO*, Directorate of veterinary Services; *RVIL*, Regional Veterinary Investigation Laboratories; *VEEU*, Veterinary Epidemiology and Economics Unit

*“Unknown” refers to cases from undefined poultry production system (i.e., production system were not recorded during the sample submission, but all the other records were availed)

## Materials and methods

### Description of poultry production systems in Kenya

The ND cases were reported from intensive, free-range, semi-housed, and caged poultry production systems **(**File S1). In the Kenyan context, intensive (or commercial) poultry is housed throughout and provided with commercial feed and veterinary care. Free-range poultry, which are left to scavenge for food and sometimes supplemented with household leftovers, may be penned at night or left outdoors to perch on trees, and veterinary services may be provided. Semi-housed chickens are allowed limited foraging time but housed at night (Martin 1992). The caged system is mainly practiced by breeders or for trading in live-bird markets.

### Description of sample origins (agro-ecological zones)

**Fig. 1:**
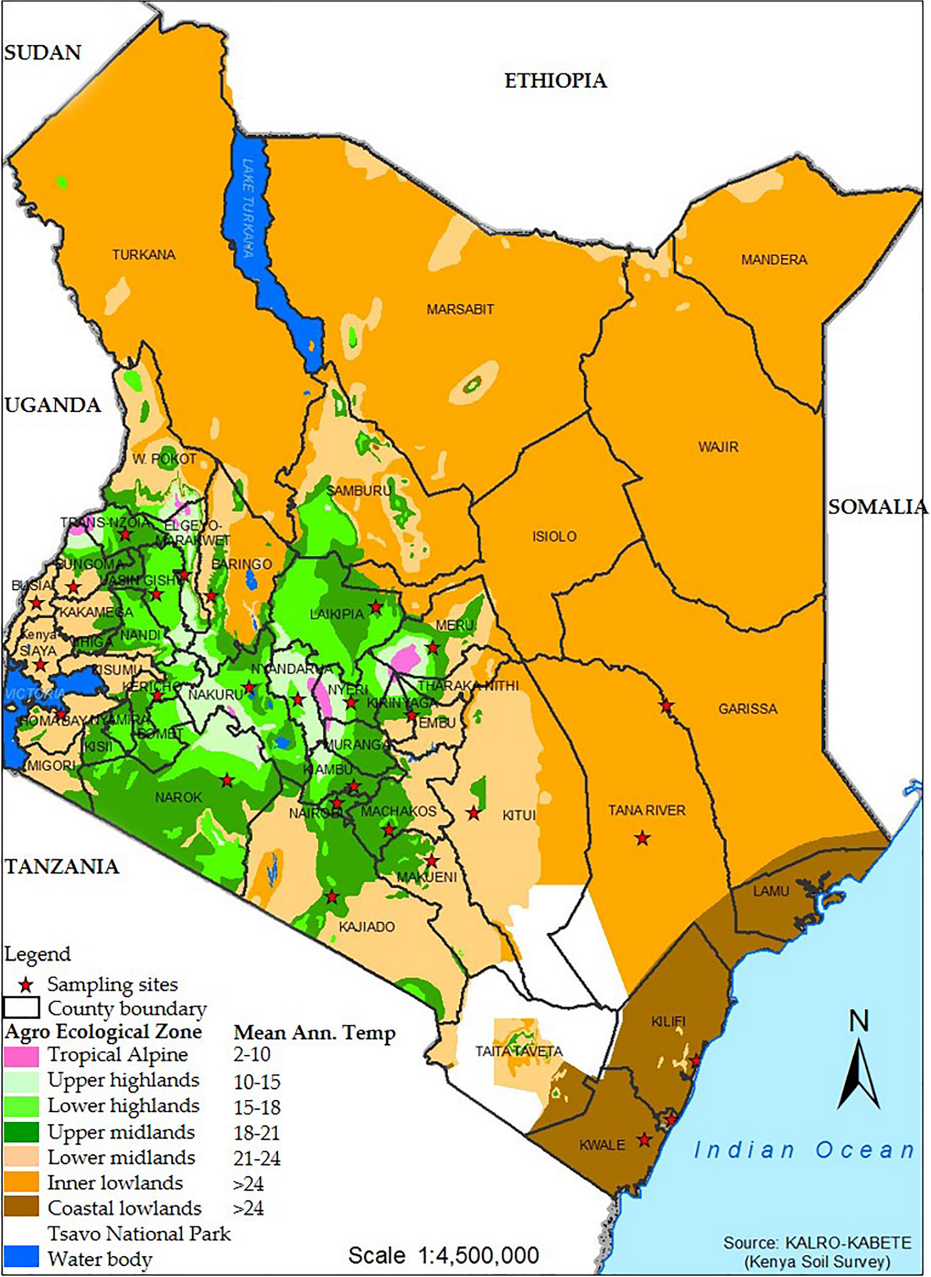
Mapping of the 27 sampling locations onto the Kenyan AEZs. The sampling sites (marked with asterisks) were distributed in six of the eight Kenyan AEZs. Samples originated from six of the eight AEZs since zones I (tropical alpine) and VIII (full desert) are not of any agricultural significance

The ND cases originated from 27 locations within six of the eight Kenyan agro-ecological zones (AEZs) (Jätzold and Kutsch 1982; Fig. 1; File S2). Briefly, zone I is largely mountains that serve as river sources, while zone VIII is a desert. Zones II (upper highlands) harbors major poultry markets and poultry farming hubs, while Zone III (lower highlands) is the most agriculturally significant AEZ containing settlement schemes. Zone IV (upper midlands) forms part of the seasonal wild bird migration route and poultry trade route. Zones V, VI, and VII (lower and inner midlands and coastal lowlands, respectively) comprise of small-scale (caged and free-range) poultry for domestic consumption. The Kenyan seasons were roughly categorized as the “dry” season (January–February), “long-rains” (March–May), “cold” (June–October), and the “short-rains” (November–December) seasons, according to the Köppen classification for tropical climates (Peel et al. 2007).

### Acquisition of climatic data from the study locations

Mean monthly temperature (°C) and percent relative humidity (RH) data were retrieved from weather stations using R language in the software RStudio version 1.1.456 (RStudio Inc., Boston, MA) (http://www.rstudio.com/). For data retrieval, weather station codes nearest the various locations and case reporting dates were entered in to the RStudio and analyzed using scripts customized from the R package weatherData version 0.5.0 (http://ram-n.github.io/weatherData/).

### ND case reports and data management

The 11-year ND data were obtained from the Kenya Directorate of Veterinary Services (DVS) and were originally derived from poultry farmers (owners), private veterinary officers, Central and Regional Veterinary Investigation Laboratories (CVL and RVILs), Veterinary Epidemiology and Economics Unit (VEEU), and County Veterinary Offices (File S2). In this study, ND outbreak duration denotes the number of days from the onset of clinical signs on specific farms, up to the time of reporting (see File S1). A reported infection was classified as a suspected ND case (hereafter referred to as “ND suspect case”) based on clinical signs and gross PM findings on tissues (e.g., liver, trachea, intestines, etc.; see File S1) consistent with ND (Alexander 2000) and history of previous ND reports from specific farms. A specific case number was assigned to each of the ND suspect cases, from which specimens were collected at necropsy and subsequently used for diagnostic tests (see File S1).

### Diagnosis of AOaV-1

Diagnostic tests were performed according to the OIE manual of Standard Diagnostic Tests (Alexander 2012). The procedures used for virus isolation in embryonated chicken eggs and HI test are described in File S2.

#### Enzyme-linked immunosorbent assay

The presence of AOaV-1 antibodies in serum samples was detected using the ProFloK® Plus NDV ELISA kit (Synbiotic Corporation, San Diego, CA) according to the manufacturer’s instructions. Briefly, 50 μl of diluted sera (1:50) was added to antigen-coated plates, incubated for 30 min at room temperature (RT), and then washed 3 times with 300 μl of wash solution. Then, 100 μl of diluted (1:100) anti-chicken IgG (H+L) conjugate was dispensed into each well and the plates incubated for 30 min at RT. After washing three times as above, 100 μl of diluted substrate solution was added to each well, followed by incubation for 15 min at RT and the reaction terminated by addition of 100 μl of stop solution to each well. Plates were read using an ELISA plate reading spectrophotometer at 405–410 nm.

#### Real-time reverse transcription polymerase chain reaction

Total RNA was extracted from allantoic fluid using TRIzol®LS protocol following the manufacturers’ recommendations. The rRT-PCR test (targeting the AOaV-1 *matrix* gene) was performed using Qiagen® one-step RT-PCR kit (Applied Biosystems, Ambion) as previously described (Wise et al. 2004), using the M+4100 forward primer (5′-AGT GAT GTG CTC GAC CTT C-3′), M-4220 reverse primer (5′ -CCT GAG GAG AGG CAT TTG CTA-3′), and M + 4169 probe (5′-FAM-TTC CTC TAG CAG TGG GAC AGC CTG C[BHQ]-3′). The composition of the rRT-PCR reaction mix (25 μl final volume) is shown in Table 2. The one-step RT step was performed for 30 min at 50 °C and 15 min at 95 °C, while the PCR thermocycling conditions for the M gene primer/probe set consisted of 40 cycles of denaturation (10 s at 94 °C), annealing (30 s at 52 °C), and extension (10 s at 72 °C).

**Table 2:** Reagents composition of rRT-PCR (25 μl final volume) for detection of AOaV-1 targeting the viral *matrix* (M) gene

Reagent	Volume(μl) per reaction	Final concentration
Nuclease-free H_2_O	6.95	
5× buffer	5	1×
25 mM MgCl_2_	1.25	3.75 mM
dNTP’s (10 mM each)	0.8	320 μM per dNTP
M + 4100 Forward primer (20 pmol/μl)	0.5	400 nM
M-4220 Reverse primer (20 pmol/μl)	0.5	400 nM
RNase inhibitor (13.3 units/μl)	0.5	0.266 units
Enzyme mix	1	
M+4169 probe (6 pmol/μl)	0.5	240 nM
Master mix per reaction	17	
Template	8	
Total reaction volume	25	

Based on the diagnostic tests described above, a case was determined as likely AOaV-1-positive (hereafter referred to as “AOaV-1-positive case”) only if the clinically sick or dead birds also tested positive with at least one of the tests, but there was no differentiation between vaccinates or natural infections. Technical variations were deemed insignificant since the same protocols were used during the entire study period.

### Statistical analyses

The Kruskal-Wallis test was used to determine differences between various sample groups (years, months, seasons, production systems, locations), followed by Dunn’s test (with Bonferroni corrections) if significant differences (*p* values of < 0.05) were observed. The correlation between climate and the ND cases were determined using the Pearson test. The statistical and correlation analyses, visualization, and plotting of the results were performed in RStudio.

## Results

### Analysis of reported ND suspect cases

During the 11-year study period, poultry owners and CVL personnel, respectively, reported approximately 42% and 41% of the 418 ND cases (Table 1). The DVO, VEEU, and private veterinarians accounted for 12.65%, 4.22%, and 3.92% of sources for the ND case reports, respectively. Of the 418 cases, 86 were without proper records and were omitted from subsequent analyses of the 332 remaining cases (File S1).

### Analyses of AOaV-1-positive cases

Of the 332 ND suspect cases, 256 (77.1%), 45 (13.6%), and 10 (3%) cases were diagnosed using virus isolation in embryonated chicken eggs, RT-PCR, and HI, respectively, either singly or in combination with PM lesions suggestive of ND (Table 3). Only 19 (5.7%) and one (0.3%) of the cases were tested by PM and ELISA, respectively. Based on these tests, only 42.16% (*n* = 140) of the 332 ND suspect cases were deemed AOaV-1-positive, of which 81, 45, and 14 cases were from the intensive, free-range, and caged systems, respectively (Table 3 and File S1). Only three cases were from affected ducks, while one case each included affected goose and turkey, all submitted as carcasses in 2006/2007 from the free-range system in urban centers (e.g., in Nairobi, Kiambu, and Nakuru). The remainder of this paper discusses only the 140 AOaV-1-positive cases.

**Table 3:** Summary of the laboratory diagnosis tests that were performed on 332 ND cases reported from 2005 to 2015 (see details in File S1)

Test performed	ND cases from different poultry production systems (numbers of AOaV-1-positive cases)					Test results (AOaV-1-positive are shown in parentheses)	
	Caged	Free-range	Intensive	Unknown*	Total	Positive	Negative
PM only	2 (2)	3 (3)	14 (14)	0 (0)	19	19 (100.0%)	0 (0.0%)
Egg isolation only	10 (3)	34 (9)	53 (18)	4 (1)	101	31 (30.7%)	73 (69.3%)
HA only	1 (1)	0 (0)	0 (0)	0 (0)	1	1 (100.0%)	0 (0.0%)
RT-PCR only	7 (1)	7 (5)	12 (4)	0 (0)	26	10 (38.5%)	16 (61.5%)
PM and egg isolation	11 (5)	48 (25)	88 (31)	8 (2)	155	63 (40.6%)	92 (59.4%)
PM and HA	2 (0)	0 (0)	7 (4)	0 (0)	9	4 (44.4%)	5 (55.6%)
PM and RT-PCR	4 (2)	2 (2)	13 (10)	0 (0)	19	14 (73.7%)	5 (26.3%)
ELISA	0	1 (1)	0	1 (1)	2	2 (100.0%)	0 (0.0%)
Total	37 (14)	95 (45)	187 (81)	13 (4)	332	144 (43.4%)	188 (56.6%)

*Unknown refers to cases from undefined poultry production system (i.e., production system were not recorded during the sample submission, but all the other records were availed)

#### Annual and seasonal variations of AOaV-1-positive cases

**Fig. 2:**
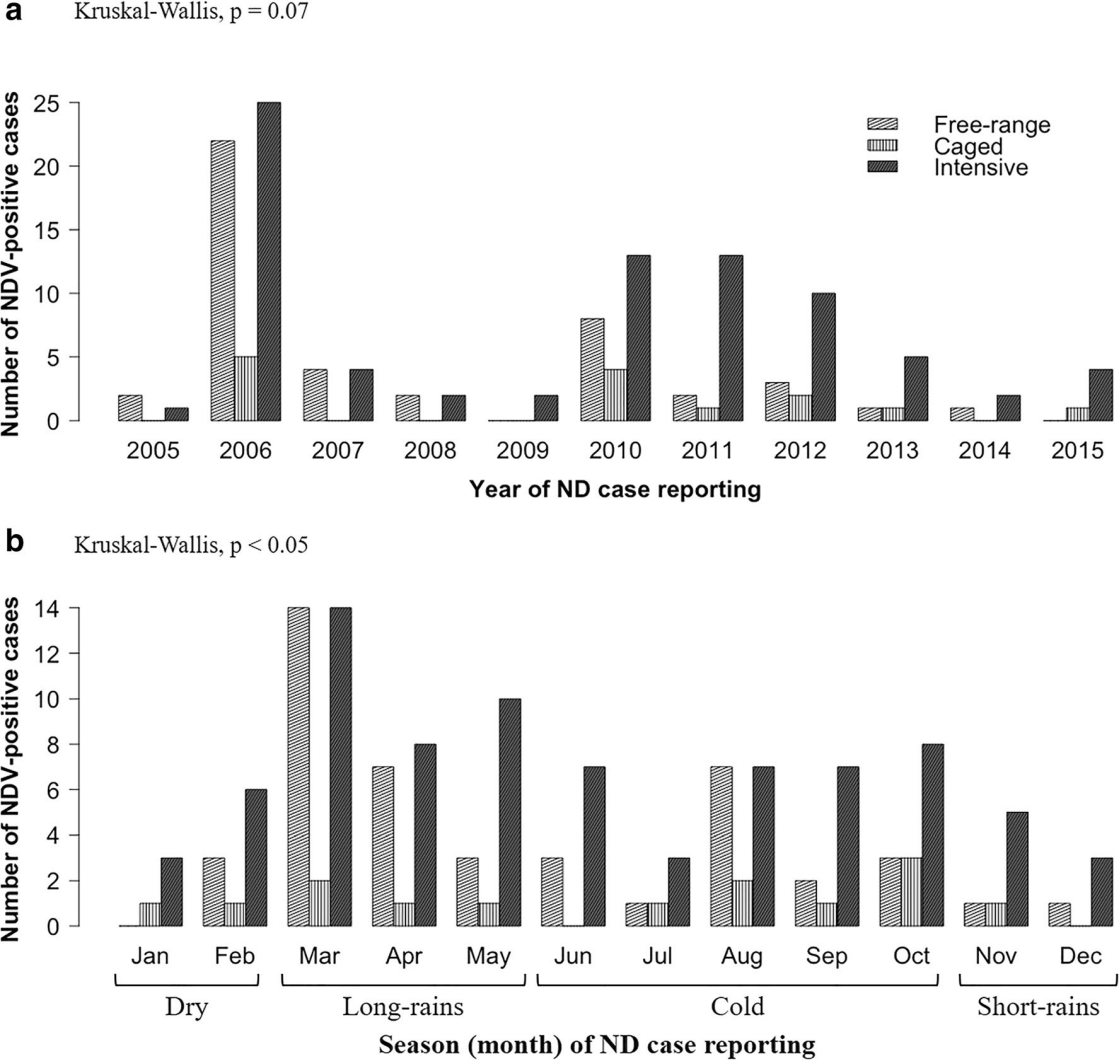
Variations in the numbers of AOaV-1-positive cases across the years (**a**) and seasons **(b**) in the free-range, caged, and intensive production systems. The cases varied more across the seasons (*p* < 0.05) compared to across the years (*p* = 0.07). The numbers of AOaV-1-positive cases were statistically different (*p* < 0.05) between the three production systems. Statistical *p* values (Kruskal-Wallis test) are indicated at the top left of the figure panels

There were wide variations in the number of AOaV-1-positive cases over the 11-year period with two high peaks in 2006 (52 cases) and 2010 (25 cases) (Fig. 2a). These variations in the numbers were statistically significant between the production systems (*p* = 0.01), but not between the years (*p* = 0.07). The intensive system experienced AOaV-1-positive cases in 11 years compared with nine and six of the years in the free-range and caged systems, respectively (Fig. 2a). Across the months, the numbers of AOaV-1-positive cases differed significantly (*p* < 0.0001) between production systems (Fig. 2b), with the intensive and the caged systems having the highest and the lowest numbers of AOaV-1-positive cases, respectively. Seasonal variations in the cases also differed significantly (*p* < 0.05) between the three systems, particularly during the long-rains and cold seasons (Fig. 2b).

#### Zonal variations of AOaV-1-positive cases

None of the 11 years nor any of the six AEZs was free of AOaV-1-positive cases. However, AOaV-1 was not diagnosed in eight of the 27 locations within the six AEZs, including one county each in zones II (Nyandarua), III (Elgeyo-Marakwet), and VI (Tana River), two locations in zone IV (Embu and Trans Nzoia), and four locations in zone V (Homa bay, Kitui, Makueni, and Siaya) (File S2). Only the free-range system experienced AOaV-1-positive cases from all the six AEZs and was highest in zones II and III (Fig. S1). Overall, the number of AOaV-1-positive cases significantly differed between locations (*p* = 0.02) and production systems (*p* = 0.04), but not between the AEZs (*p* = 0.09). Only four of the 19 locations reported AOaV-1-positive cases from all three productive systems, i.e., Nairobi (zone II), Kericho and Nakuru (zone III), and Kiambu (zone IV).

### Analyses of the numbers of clinically sick and dead birds

Each of the ND cases affected multiple birds, which were submitted at the CVLs either alive (clinically sick) or dead. Therefore, comparative analyses were performed to determine variations of these numbers in the three poultry production systems across the months and seasons of the 11-year study period.

#### Annual variations in the numbers of affected birds

**Fig. 3:**
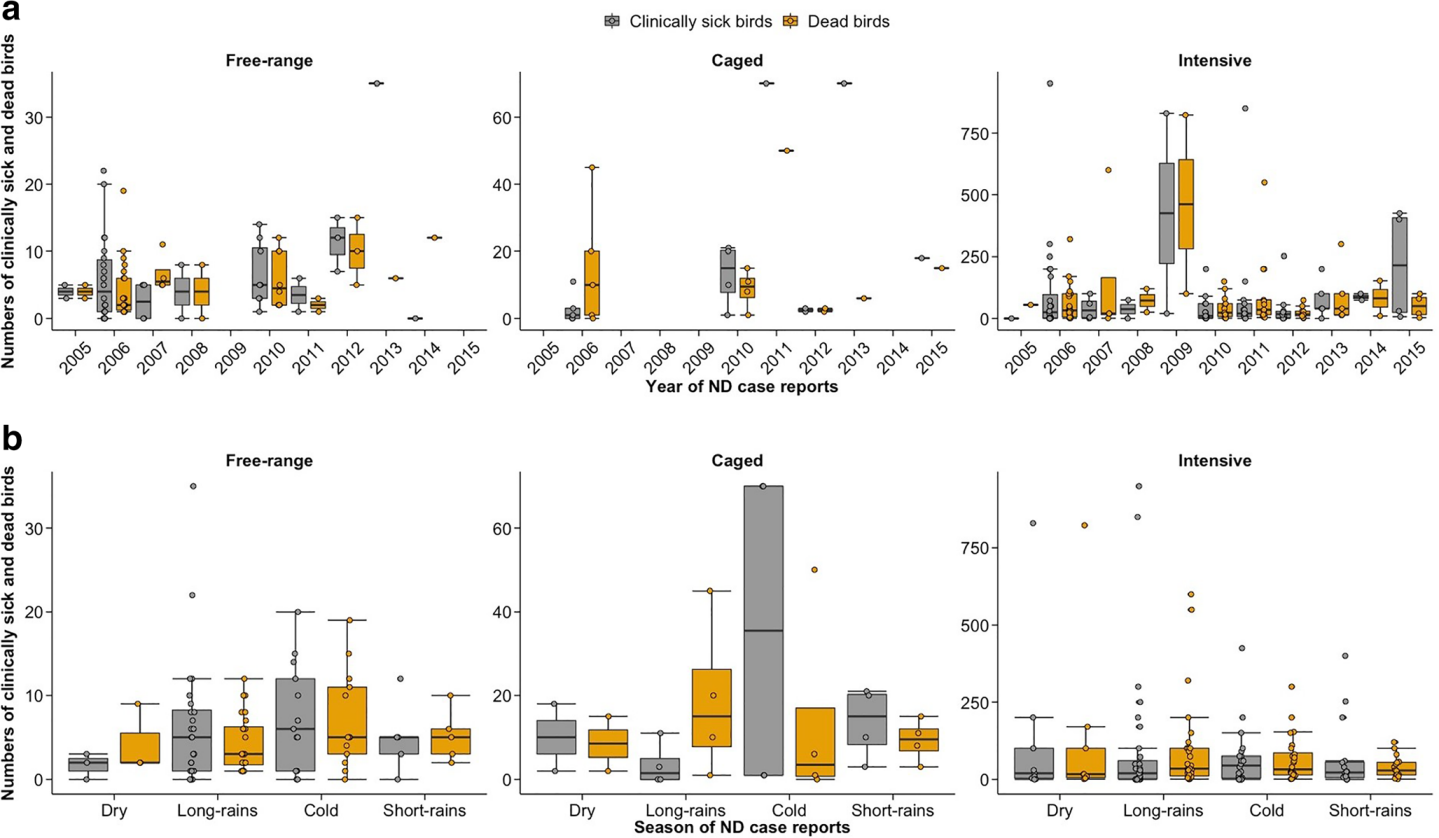
Variations in the numbers of clinically sick and dead birds across the years (**a**) and seasons (**b**) in the free-range, caged, and intensive productive systems. The numbers of affected birds depended on the productive system, with intensive system represented in every year (**a**). During the cold season, birds’ mortalities exceeded the clinically sick birds in the intensive system (**b**). Similar trends are apparent the long-rain season in the caged system, the dry and short-rain seasons in the free-range system (**b**)

Overall, the total numbers of clinically sick birds were significantly higher (*p* < 0.001) than mortalities reported (Fig. 3). However, in five out of the 11 years (2007–2010 and 2013), mortalities exceeded clinically sick birds in specific production systems, for example in the intensive system (2007–2010 and 2013), caged system (2006), and free-range system (2007 and 2014) (Fig. 3a). Despite the intensive system recording the highest numbers of clinically sick birds throughout the 11-year period, in more than 50% of the study period (i.e., in 2007, 2008, 2010, and 2011–2013), the percentage of the flocks with manifestation of ND clinical signs were considerably lower compared with the caged (in 2010, 2011, and 2013) and free-range (in 2008 and 2010–2013) systems (Fig. 3a). In 2011 and 2013, up to 70 to 100% of the reported cases from the caged system presented descriptions as either clinically sick or dead (see Fig. 3a).

#### Seasonal variations in the numbers of affected birds in different production systems

Like the trend observed across the 11-year study period, the reported mortalities exceeded clinically sick birds depending on seasons and production system (Fig. 3b). Higher numbers of mortalities exceeded numbers of clinically sick birds during the cold season in the intensive system, the long-rains season in the caged system and during the dry and short-rains seasons in the free-range system (Fig. 3b).

### Correlation between climate and numbers of ND cases

**Fig. 4:**
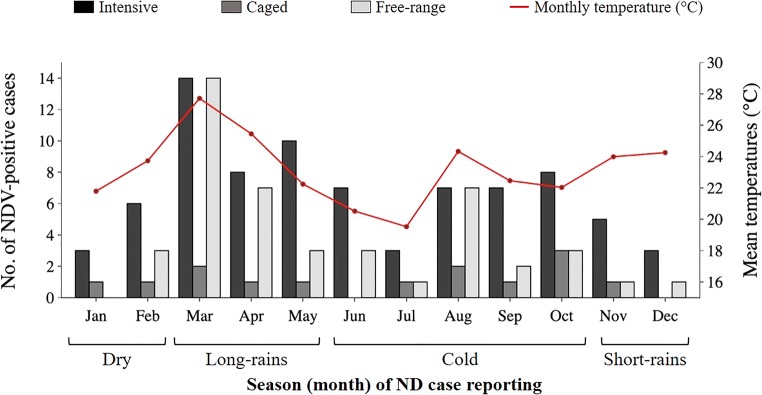
Relationship between mean monthly temperatures and numbers of AOaV-1-positive cases. A peak in the numbers of AOaV-1-positive cases is notable during months with higher temperatures (March and August) compared with the cold season (June/July)

Figure 4 presents seasonal variations of the numbers of AOaV-1-positive cases in different poultry production systems. The numbers of AOaV-1-positive cases were significantly affected by the type of poultry production system (*p* < 0.001), ambient temperatures (*p* = 0.001), and season (*p* = 0.003), but not by the relative humidity (RH; *p* = 0.7). The effects of seasons and climate on the numbers of AOaV-1-positive cases differed from one production system to another. For example, in the free-range system, both the temperatures and season had the most significant effects (*p* = 0.001 and 0.02, respectively), while in the intensive system and caged systems, cases were significantly affected by the season and RH, respectively (*p* = 0.05).

There was a linear positive linear correlation between the numbers of AOaV-1-positive cases and the temperatures and RH (correlation coefficient, *r* = 0.8 and 0.5, respectively) in the free-range system, but weak correlation in the intensive (*r* = 0.1 and 0.2, respectively) and caged systems (*r* = 0.4 and 0.2, respectively). Considering the combined effects of the type of production system, season, and climate, the numbers of AOaV-1-positive cases were significantly associated with the combined effects of production system and the ambient seasonal temperatures (*p* < 0.05). Finally, the numbers of clinically sick birds were significantly related to the ambient seasonal temperatures (*p* < 0.05), while mortalities significantly associated with the season (*p* < 0.05). In all three production systems, the correlation between the numbers of clinically sick birds and temperatures was strongly linear (*r* = 0.6), but the correlation was relatively weak with RH (*r* = 0.4).

### Times of ND reporting

Respectively, ~ 82% and 90% of the caged and free-range farmers reported cases within 7 days of observing clinical signs, while the rest reported after 30–90 days. Only ~ 62% of the intensive farmers reported within 7 days, while the rest reported after 14 days (~ 16%), 30 days (~ 13%), or 60–90 days (~ 9%). Farmers from zone IV took significantly longer to report cases compared with the famers from zones II and III (*p* values of 0.04 and 0.01, respectively). Farmers generally took longest to report cases from the end of the long-rains (May) to cold (June and August–September) seasons.

## Discussion

The continued presence of AOaV-1-positive cases in all 11 years and in all six AEZs agrees with earlier studies that showed occurrence of ND outbreaks once or twice a year at regular intervals in Kenya (Awan et al. 1994). This trend suggests endemicity of virulent AOvA-1 in Kenya, particularly in the free-range system, which contributes significantly to the rural economy in the country (Magothe et al. 2012). Unfortunately, during the study period reported here, serological surveys (e.g., detection of antibodies against AOaV-1) to determine exposure of unvaccinated free-range chickens to natural AOaV-1 infections or to test the efficiency of vaccinations in chickens reared under intensive system were not available.

Reporting, diagnosis, and follow-up of ND outbreaks in Kenya were non-uniform. Almost 40% of the reported ND cases originated from farmers seeking ND confirmation, which highly depends on the proximity of the farmers to the CVLs, thus explaining the wide variations in the ND case reports between specific locations, especially the urban centers in AEZs II, III, and IV. Veterinary personnel reported a further 41% of the cases during occasional surveillance/vaccination campaigns, which may not have extensive coverage, particularly in the interior rural areas. The proportion of ND suspect cases that diagnostically tested AOaV-1-positive was considerably lower than expected; i.e., only ~ 43% of the cases suspected to be ND were positive. It is possible that the clinically sick or dead birds in most of the ND suspect cases suffered from infections with other agents (mistaken to be due to ND), or that virus strains were undetectable (non-hemagglutinating or mutants) by the diagnostic methods used in this study (Dimitrov et al. 2016).

The time for ND reporting during the period covered in the current study appears dependent on the type of production system; 56%, 28% and 11% accounted for the cases reported from the intensive, free-range, and caged systems, respectively. The intensive famers represent a minority of the Kenyan poultry industry, but with the highest numbers of ND case reports. However, in terms of the time taken by farmers to report the cases to the CVLs, the caged and free-range farmers appear to report the cases faster and more frequently than the intensive farmers do. A possible explanation for this practice is that the intensive farmers take precautions (e.g., scheduled vaccinations or preventive/curative medicines, separation of sick birds, etc.), which may negate immediate reporting to the CVLs.

Poultry population in Kenya is estimated at 32 million, of which ~ 26 million are free-ranging indigenous chickens or crosses with exotic breeds, while the rest are commercial broilers and layers kept under the caged and intensive (commercial) systems (Onono et al. 2018). The 6 million chickens under the intensive production system are the chickens that are vaccinated under the three-round vaccination regime used in Kenya. Most of the small-scale farmers do not vaccinate their flocks and are not even aware that ND can be controlled by vaccination (Kingori et al. 2010). Furthermore, the existing NDV vaccination regimes in Kenya are not well enforced to provide a protective flock-level immunity, which is achieved only when high proportions (≥ 85%) of birds have hemagglutination inhibition antibody titers ≥ 8 to NDV 8 (based on 8 HAU/50 μl of antigen) (van Boven et al. 2008). Other ND control measures in Kenya include enhanced biosecurity and restriction on movement of poultry in the country. However, Kenya lacks government-sponsored programs to support ND eradication via culling or compensation policies, a deficiency that has also been reported in Latin America (Absalón et al. 2019). Thus, farmers have different ways of handling sick birds in their flocks. For instance, majority of the commercial farmers quarantine the sick birds and seek advice from veterinarians on treatment options compared to the small-scale farmers who slaughter sick birds before they die (for sale or home consumption) (Onono et al. 2018). These differences in vaccination and control measures between commercial and small-scale farmers may partially account for the trends in disease reporting in Kenya reported in this paper.

Detection of AOaV-1-positive cases from all the six AEZs by free-range farmers shows the importance of this system to ND epidemiology. The strong linear correlation between ND cases and climate from this study in the free-range system implicates this system as likely to be an important player in the ND epidemiology in rural poultry. Accounting for ~ 84% of chicken, the free-range system is almost entirely based on low-input/output management with little attention to poultry health (Ashraf and Shah 2014; Magothe et al. 2012). Consequently, most small-scale (free-range and caged) farmers may not prioritize disease reporting. Upon noticing ND clinical signs, some of the small-scale farmers consume the sick chickens or quickly sell off their flocks to unsuspecting traders (due to anticipated birds’ mortalities) (Njagi 2008; Nguyen 1992; Musiime 1992). Other farmers treat their birds using inefficient traditional plant-based herbs, and therefore the sick birds linger around the neighborhoods as the farmers wait for their recovery. Introduction of such birds into other susceptible flocks could contribute to disease spread due to their scavenging nature (Spradbrow 1993; Kuiken et al. 1998; Njagi et al. 2012; Elrom 2000; Heuschele and Easterday 1970; Utterback and Schwartz 1973), which could explain the high proportions of flocks showing clinical signs in the caged and free-range systems compared with the intensive sysyem.

The finding of high mortalities under the intensive system points to additional problems associated with confinement, especially under poor biosecurity conditions (Njagi 2008), which may influence disease outbreaks. In fact, penning birds indoors during the cold weather may also explain our findings of high numbers of clinically sick and dead birds in the intensive and the caged systems during the long-rains and cold seasons. Sudden weather changes could influence viral transmission, and thus the timing and the intensity of disease outbreaks, which correlate with alternations of heavy rains, drought, elevated temperatures, and humidity (Martin 1992; Awan et al. 1994; Nyaga et al. 1985; Mukiibi 1992; Njagi et al. 2010; Hines and Miller 2012; Nayak et al. 2015).

The finding of relatively low numbers of AOaV-1-positive cases from caged and free-range farmers during the short rains and dry seasons may reflect the relationship between culturally significant periods (e.g., festivities) and ND outbreaks and/or reporting. For example, small-scale farmers sell off their flocks immediately before the onset of the dry season (coinciding with high poultry demand during Christmas and New Year festivities) during which AOaV-1 may be under incubation (Musiime 1992; Mukiibi 1992). In addition, during such periods, some commercial farmers, who regularly vaccinate their flocks against ND, sell off their “spent” chickens to small-scale farmers. Such birds may mingle with potentially susceptible free-range birds (Martin 1992), which may explain the high AOaV-1-positive cases observed during the subsequent long-rains seasons in the free-range and the caged systems. Such disease dynamics may be dependent on specific AEZs (Njagi 2008) perhaps due to differences in seasonal and poultry management. Our finding of more AOaV-1-positive cases at the start of the long-rains and mid-cold seasons, especially in some key live-bird markets and trade routes in zone II and IV, agrees with many previous studies (Thitisak et al. 1988; Musiime 1992; Martin and Spradbrow 1992).

Despite gaining significance in Kenya, duck, turkey, pigeon, ostrich, guinea fowl, and quail, which gregariously mingle with domestic chickens, do not appear to be significantly affected by ND. Data from the current study appear to suggest that most of the households who keep these species do not report ND cases, possibly accounting for the relatively low numbers of ND case reports on these species. This is with the exemption of a spike in the submission of cases (> 85%) affecting duck, turkey, and geese in 2006, which coincided with an avian influenza scare in Kenya (October 2005 to mid-2006) (Nyaga 2008; Matheka et al. 2013). It is also possible that the low numbers of cases from these species could be due to their covert AOaV-1 infections and/or resistance AOaV-1 (Njagi et al. 2012; Dai et al. 2014); hence, farmers may not report cases for lack of clinical signs. Nevertheless, even when these other species do not develop clinical signs, they may still shed virus, which acts as source of infections for domestic chickens through feces, implements (e.g., feeders, drinkers, and cages), contaminated feeds, etc., (Martin 1992; Sharif et al. 2014).

In conclusion, the current study indicates that AOaV-1 is endemic and widely distributed in Kenya. The study also shows the need for improved ND management in Kenya, as evidenced by the lack of comprehensive serological and diagnostic tests to confirm presence of AOaV-1 variants in the analyzed samples, and the slow and incomplete reporting system. Among the key improvements needed include implementation of government-sponsored prevention and control strategies such as active surveillance programs, enhanced biosecurity, strict vaccinations regimes, and creating awareness about these measures. The positive correlation between climate and cases could be exploited in the development of a more robust active serological surveillance of ND in Kenya, especially in unvaccinated rural free-range chickens. Further studies are needed to identify and characterize the circulating AOaV-1 genotypes, determine their transmission risks, which are key factors in development of improved disease diagnostics, surveillance, and prevention programs.

## Electronic supplementary material

File S1 **Fig. S1**: - The reporting lines within the disease surveillance in Kenya. The submitters of the ND cases/samples used in the current study are highlighted. **Table S1**: - Details of the 418 cases reported from different locations from 2005 to 2015 covered in this study. **Table S2**: - 332 cases selected from the case records for further analyses based on the completeness of the available details. **Table S3**: - 140 reported that tested probable-positive for ND infections when subjected to various serological and/or virological tests (see manuscript text for details). (XLSX 241 kb)

File S2 **(A)** Description of the origin of the samples used in the current study (**Table S1**). The AEZs are land resource mapping units with specific similar characteristics in terms of the agricultural suitability, potential and constraints for production, and environmental impacts. The AEZs are based on FAO (1996) zoning (Agro-ecological zoning guidelines, FAO soils Bulletin 73; mailto:http://www.fao.org/docrep/W2962E/W2962E00.htm). (B) Laboratory diagnosis of AOaV-1 by: (i) isolation in embryonated eggs; (ii) hemagglutination-inhibition (HI) assay tests; (iii) enzyme-linked immunosorbent assay (ELISA); and (iv) reverse transcription-polymerase chain reaction (RT-PCR). (DOCX 30 kb)

Figure S1 **Variations of the AOaV-1- positive cases within the six Kenyan AEZs**. The differences in the numbers of AOaV-1- positive cases were not significantly between the AEZs (*p* = 0.09). The most significant difference was between the intensive and the caged system, particularly zone IV. (JPG 60 kb)
