# Supplementary material for: A retrospective study of Newcastle disease in Kenya
Source: Trop Anim Health Prod. 2019 Sep 10;52(2):699–710. doi: 10.1007/s11250-019-02059-x (PMC7039849; doi:10.1007/s11250-019-02059-x)
Supplement: Supplementary file 2 — (A) Description of the origin of the samples used in the current study (Table S1). The AEZs are land resource mapping units with specific similar characteristics in terms of the agricultural suitability, potential and constraints for production, and environmental impacts. The AEZs are based on FAO (1996) zoning (Agro-ecological zoning guidelines, FAO soils Bulletin 73; mailto:http://www.fao.org/docrep/W2962E/W2962E00.htm). (B) Laboratory diagnosis of AOaV-1 by: (i) isolation in embryonated eggs; (ii) hemagglutination-inhibition (HI) assay tests; (iii) enzyme-linked immunosorbent assay (ELISA); and (iv) reverse transcription-polymerase chain reaction (RT-PCR). (DOCX 30 kb) [file 11250_2019_2059_MOESM2_ESM.docx]

1. **Table S1:** Description of the origin of the samples used in the current study. The AEZs are land resource mapping units (climate, land reforms, soils, and land cover) with specific similar characteristics in terms of the agricultural suitability, potential and constraints for production, and environmental impacts. The AEZs are based on FAO (1996) zoning (Agro-ecological zoning guidelines, FAO soils Bulletin 73; <http://www.fao.org/docrep/W2962E/W2962E00.htm>).

| **Location**^*^ | **GPS Coordinates** | | | **Description of the Agro ecological zone (AEZ)** | | |
| --- | --- | --- | --- | --- | --- | --- |
|  | **latitude** | **longitude** | **elevation (m)** | **name** | **annual mean temp & rainfall** | **remarks on farming and/or poultry production** |
| Nairobi | -1.2921 | 36.8219 | 1656 | **Zone II**:  (Upper highlands) | 10 -15°C;  > 1000 mm | - Poultry faming is staple food and income source - Major poultry markets |
| Nyandarua^*^ | -0.3994 | 36.4895 | 2409 |  |  |  |
| Nyeri | -0.4278 | 36.9434 | 1822 |  |  |  |
| Elgeyo-Marakwet^*^ | 0.6726 | 35.508 | 2351 | **Zone III**:  (Lower highlands) | 15-18°C  950 - 1500 mm | - The most agriculturally significant Kenyan zone - Most human resettled zone in Kenya - Crop-livestock systems practiced |
| Kericho | -0.3673 | 35.2813 | 1979 |  |  |  |
| Meru | 0.0515 | 37.6456 | 1643 |  |  |  |
| Nakuru | -0.2964 | 36.069 | 1810 |  |  |  |
| Uasin Gishu | 0.5143 | 35.2697 | 2063 |  |  |  |
| Baringo | 0.4897 | 35.7412 | 2025 | **Zone IV**:  (Upper midlands) | 18-21°C;  500-1000 mm | - Part of seasonal (wild) bird emigrational routes - Major trade route for poultry |
| Embu^*^ | -0.5388 | 37.4596 | 1302 |  |  |  |
| Kajiado | -2.0981 | 36.782 | 1570 |  |  |  |
| Kiambu | -1.1462 | 36.9665 | 1507 |  |  |  |
| Laikipia | 0.397 | 37.1588 | 1828 |  |  |  |
| Machakos | -1.5177 | 37.2634 | 1611 |  |  |  |
| Narok | -1.0875 | 35.8771 | 1867 |  |  |  |
| Trans Nzoia^*^ | 1.0219 | 35.0015 | 1897 |  |  |  |
| Bungoma | 0.5695 | 34.5584 | 1431 | **Zone V**:  (Lower midlands) | 21-24°C  300-600 mm | - Caged and free-range small-scale poultry farming (protein source) |
| Busia | 0.4347 | 34.2422 | 1178 |  |  |  |
| Homabay^*^ | -0.5287 | 34.4594 | 1182 |  |  |  |
| Kitui^*^ | -1.3751 | 37.9952 | 1114 |  |  |  |
| Makueni^*^ | -1.7865 | 37.6311 | 1121 |  |  |  |
| Siaya^*^ | -0.0998 | 34.2747 | 1246 |  |  |  |
| Garissa | -0.4532 | 39.6461 | 146 | **Zone VI**:  (Inner lowlands) | >24°C  300-450 mm | - Small-scale poultry keeping at household level (source of livelihood) - Poultry is the second-most main agricultural activity; undertaken mainly by women |
| Tana River^*^ | -1.5868 | 39.4424 | 186 |  |  |  |
| Kilifi | -3.5107 | 39.9093 | 6 | **Zone VII**:  (Coastal lowlands) | >24 °C  150-350 mm | - Small-scale poultry keeping |
| Kwale | -4.1816 | 39.4606 | 362 |  |  |  |
| Mombasa | -4.0147 | 39.6918 | 47 |  |  |  |

^*^ The cases from the nine locations marked with asterisks did test positive for AOaV-1 infections

1. **Diagnosis of avian orthoavulavirus-1 (AOaV-1)**

The following procedures for virus isolation and hemagglutination inhibition (HI) assays were performed according to the OIE manual of Standard Diagnostic Tests (Alexander, 2012).

1. *Isolation of AOaV-1 using embryonated eggs*

Virus isolation was carried out from different specimens (e.g. tracheal/oropharyngeal/cloacal swabs, lungs, kidneys, intestines; etc.; see **Supplementary File S1** for more details), which were processed separately and/or in pools Briefly, suspensions in antibiotic solutions prepared from tracheal/oropharyngeal/cloacal swabs obtained from live birds, or of pooled organ samples taken from dead birds, were inoculated into the allantoic cavity of inoculated into 9-10-day old specific pathogen-free embryonated chicken eggs. The eggs were incubated (37°C; candled twice daily) for 4-7 days. Embryos that died or survived at the end of the incubation period were chilled (2 h; 4°C), at which time the allantoic fluids was harvested and stored (-70°C) in sterile, screw-capped vials until they were tested for hemagglutinating activity.

1. *Detection of AOaV-1 by hemagglutination-inhibition assay (HI) tests*


After harvesting, the allantoic fluid was subjected to standard HI assay (in duplicates) using V-bottomed microtiter plates (75 µl final volume), which was performed according to the manual off standards for OIE’s diagnostic tests and vaccines. Briefly, two-fold close dilutions of each sample (25 µl) was made across the plates, followed by addition of four hemagglutination assay units (HAU) virus/antigen and 25 µl of 1% (v/v) chicken RBC suspensions. Controls included PBS, 1% RBCs, and specific virus-positive and negative sera. The highest dilution of serum causing complete inhibition of four HAU of antigen was taken as the HI titer. Agglutination was assessed by tilting the plates and only the wells in which RBCs streamed at the same rate as the control wells were considered to show inhibition. The validity of the results was assessed against negative and positive control sera according to standard procedures.

**References**

Alexander, D.J., 2012. Newcastle disease (infections with Newcastle disease virus). Chapter 2.3.14. Newcastle Disease. In*, B. Manual of Diagnostic Tests and Vaccines for Terrestrial Animals*, 2012, 7^th^ ed*.* World organization for Animal Health (OIE): Paris-France, pp 555–573.
