# Supplementary figures and images for: A retrospective study of Newcastle disease in Kenya
Source: Trop Anim Health Prod. 2019 Sep 10;52(2):699–710. doi: 10.1007/s11250-019-02059-x (PMC7039849; doi:10.1007/s11250-019-02059-x)

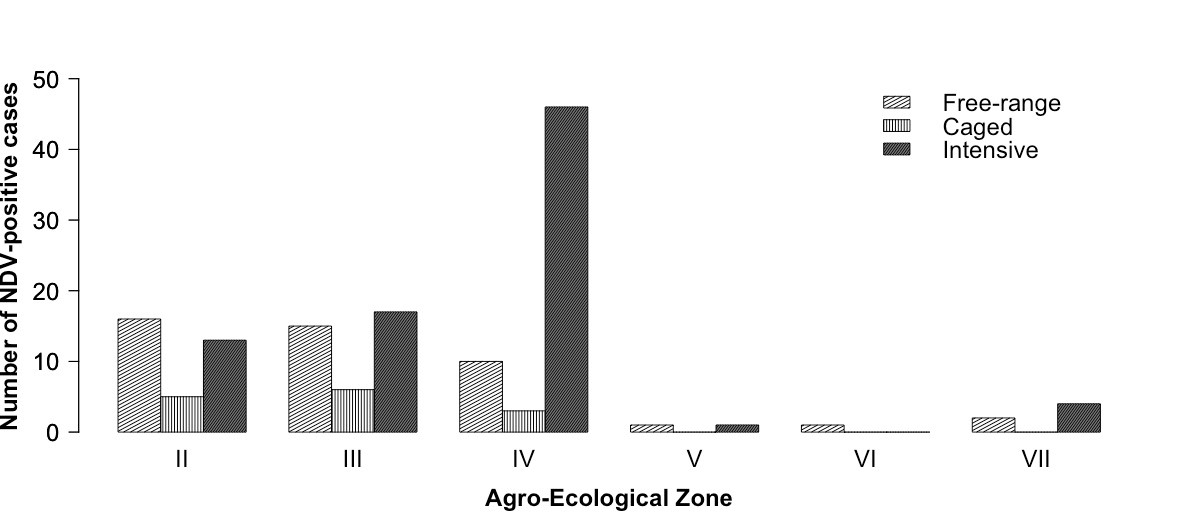

Supplement: Supplementary file 3 — Variations of the AOaV-1- positive cases within the six Kenyan AEZs. The differences in the numbers of AOaV-1- positive cases were not significantly between the AEZs (p = 0.09). The most significant difference was between the intensive and the caged system, particularly zone IV. (JPG 60 kb) [file 11250_2019_2059_MOESM3_ESM.jpg]
